# Multi-site-mediated entwining of the linear WIR-motif around WIPI β-propellers for autophagy

**DOI:** 10.1038/s41467-020-16523-y

**Published:** 2020-06-01

**Authors:** Jinqi Ren, Ruobing Liang, Wenjuan Wang, Dachuan Zhang, Li Yu, Wei Feng

**Affiliations:** 1 National Laboratory of Biomacromolecules, CAS Center for Excellence in Biomacromolecules, Institute of Biophysics, Chinese Academy of Sciences, 15 Datun Road, 100101 Beijing, China; 2 College of Life Sciences, University of Chinese Academy of Sciences, 100049 Beijing, China; 3 School of Life Sciences, Tsinghua University, 100084 Beijing, China

**Keywords:** Structural biology, Autophagy, X-ray crystallography

## Abstract

WIPI proteins (WIPI1-4) are mammalian PROPPIN family phosphoinositide effectors essential for autophagosome biogenesis. In addition to phosphoinositides, WIPI proteins can recognize a linear WIPI-interacting-region (WIR)-motif, but the underlying mechanism is poorly understood. Here, we determine the structure of WIPI3 in complex with the WIR-peptide from ATG2A. Unexpectedly, the WIR-peptide entwines around the WIPI3 seven-bladed β-propeller and binds to three sites in blades 1–3. The N-terminal part of the WIR-peptide forms a short strand that augments the periphery of blade 2, the middle segment anchors into an inter-blade hydrophobic pocket between blades 2–3, and the C-terminal aromatic tail wedges into another tailored pocket between blades 1–2. Mutations in three peptide-binding sites disrupt the interactions between WIPI3/4 and ATG2A and impair the ATG2A-mediated autophagic process. Thus, WIPI proteins recognize the WIR-motif by multi-sites in multi-blades and this multi-site-mediated peptide-recognition mechanism could be applicable to other PROPPIN proteins.

## Abstract (web summary)

WIPI proteins are mammalian PROPPIN family phosphoinositide effectors that recognize a WIPI-interacting-region (WIR)-motif to recruit other regulators during autophagosome formation. Here, the authors combine structural and functional studies and present the crystal structure of human WIPI3 with a bound ATG2A WIR-peptide and observe that the WIR-motif binds to three sites in blades 1-3 of WIPI3 and mutating these binding sites impairs the ATG2A-mediated autophagic process.

## Introduction

Autophagy is a lysosome-mediated catabolic process for the clearance of damaged organelles or protein aggregates to maintain cellular homeostasis^1–3^. Macroautophagy (hereafter, “autophagy”) is characterized by a double-membrane structure termed autophagosome that captures a variety of cellular components and delivers them to lysosome for degradation^4^. Formation of autophagosome at intracellular membrane contact sites strictly depends on phosphatidylinositol 3-phosphate (PI(3)P) that can be decoded by a number of specific downstream effectors^5,6^. These phosphoinositide effectors cannot only sense the PI(3)P signal but also act as scaffolds to recruit other proteins or protein complexes for the progression of autophagosome formation^7–9^. Mutations of these proteins severely impact on the autophagic process and are also intimately coupled with diseases such as cancers and neuronal disorders^10–12^.

PROPPIN (β-propellers that bind polyphosphoinositides) proteins are phosphoinositide effectors that play prominent roles in autophagosome biogenesis^13,14^. There are three PROPPINs in yeast (Atg18, Atg21, and Hsv2) and four in mammals (also termed WIPI (WD-repeat protein interacting with phosphoInositides), WIPI1 to WIPI4)^15,16^. PROPPINs adopt a seven-bladed β-propeller fold, in which the two neighboring sites in blades 5–6 contribute to the binding of phosphoinositides^17–19^. PROPPINs can also function as scaffolds to integrate the phosphoinositide signaling with other regulators for controlling autophagy. In mammals, WIPI4 forms a complex with ATG2A/B that may act as a tethering factor for autophagosome expansion^20,21^, while WIPI2 interacts with ATG16L1 for the efficient recruitment of the ATG12-ATG5-ATG16L1 complex and the lipidation of LC3^9^. Similar scaffolding functions were reported for yeast PROPPINs Atg18 and Atg21, respectively^8,22–24^. In addition, WIPI3 and WIPI4 can act as scaffolds to further integrate the LKB1-AMPK-TSC signaling for controlling the autophagic process^7^. However, the mechanism underlying the scaffolding role of PROPPINs and their recognition of target proteins remains largely unclear.

Among four WIPI proteins, WIPI3 and WIPI4 specifically recognize a linear characteristic peptide sequence from ATG2A/B^20,21^. We denote this linear peptide sequence as the WIR (WIPI-interacting-region)-motif. In this study, we determine the structure of WIPI3 in complex with the WIR-peptide from ATG2A. Unexpectedly, the WIR-peptide wraps around the WIPI3 β-propeller and binds to three sites in blades 1–3. Based on the WIR-peptide complex structure, we define the peptide-binding sites in WIPI proteins and the signature WIR-motif in their target proteins. Mutations in these peptide-binding sites disrupt the interactions between WIPI3/4 and ATG2A and impair the ATG2A-mediated autophagic process. This work reveals the multisite-mediated peptide-recognition mechanism of WIPI β-propellers, which could be extended to other PROPPIN family members.

## Results and discussion

### WIPI-interacting region in ATG2A for binding to WIPI3

To investigate the mechanism underlying the recognition of the linear WIR-motif by WIPI proteins, we initiated this work with characterizing the interactions between WIPI proteins and the WIPI-binding fragments of ATG2A. Although both WIPI3 and WIPI4 were found to interact with ATG2A^20^, WIPI3 exhibited excellent protein sample quality and was chosen for biochemical and structural studies^25^. On the other hand, the WIPI-interacting region in ATG2A was narrowed down to residues 1358–1404^21^. Based on the primary sequence analysis of ATG2, we removed the N-terminal 15 residues from this region and used the two fragments of ATG2A (the long fragment (1358–1404) and short fragment (1374–1404), referred to as ATG2A^long^ and ATG2A^short^, respectively) tagged with GB1 for the isothermal titration calorimetry (ITC) assay (Supplementary Fig. 1a, b). As the control, GB1 alone did not bind to WIPI3, but both ATG2A^long^ and ATG2A^short^ bound to WIPI3 with a similar affinity (with the Kd of 8.6 ± 0.1 μM and 8.7 ± 0.1 μM, respectively) (Supplementary Fig. 1c), indicating that the N-terminal 15 residues of ATG2A^long^ is not involved in the binding to WIPI3. Thus, ATG2A^short^ could be the minimum WIPI-interacting region in ATG2A for binding to WIPI3 and was referred to as the WIR-peptide.

### Crystallization of WIPI3 in complex with the WIR-peptide from ATG2A

**Fig. 1:**
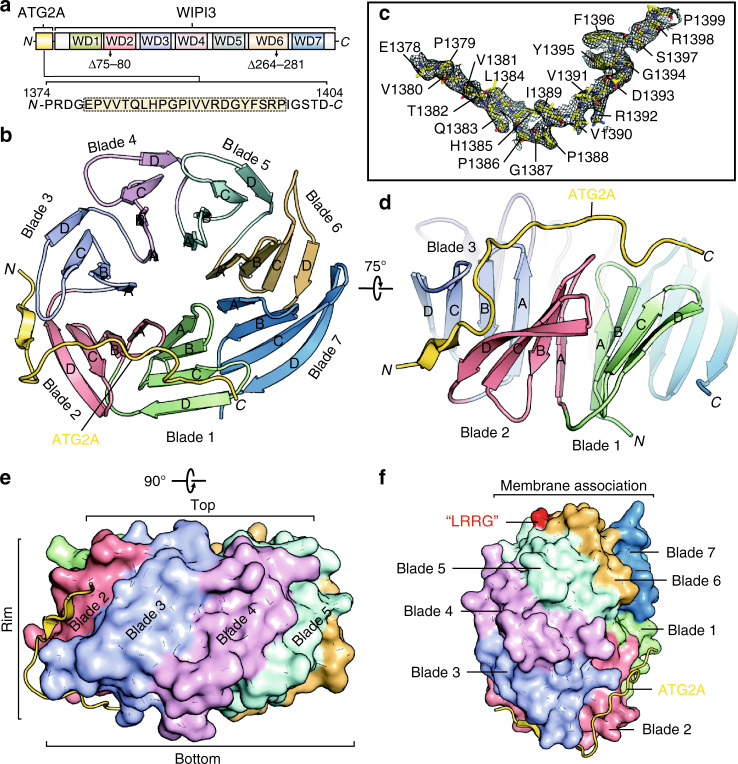
Overall structure of the WIPI3-Δloop/WIR-peptide complex. **a** The fusion construct used for crystallization. **b**–**d** A ribbon diagram of the WIPI3-Δloop/WIR-peptide complex structure from a bottom (**b**) and a side (**d**) view. Each blade of WIPI3 and the WIR-peptide from ATG2A are colored follows the color schemes in **a**. The omit electron density map of the WIR-peptide is shown and contoured at 1.0 σ level (**c**). **e**–**f** A combined surface and ribbon diagram showing the disk-like structure of the complex. The WIR-peptide wraps around WIPI3 from the rim to bottom and is distal from the membrane-binding site.

Next, we tried to crystallize this WIPI3/WIR-peptide complex, but full-length WIPI3 tends to aggregate and failed to be crystallized. Fortunately, a loop-deletion mutant of WIPI3 (referred to as WIPI3-Δloop), with deletions of two flexible loops (Blade 2 B–C loop (75–80) and Blade 6 C–D loop (264–281)), shows enhanced protein sample quality and has been crystallized for structural determination^25^. To evaluate the potential effect of loop deletions on the WIR-peptide interaction, we measured the binding affinities between WIPI3-Δloop and the two fragments of ATG2A. These two fragments bind to WIPI3-Δloop with the similar affinities to that of wild-type WIPI3 (with the Kd of 6.3 ± 0.2 μM and 6.4 ± 0.7 μM, respectively) (Supplementary Fig. 1d), suggesting that deletions of two loops have little impact on the binding. Thus, WIPI3-Δloop was chosen for crystallization in complex with the WIR-peptide. However, we did not get diffraction-quality crystals by co-crystallization. We then adopted the fusion strategy by which the WIR-peptide is fused to either the N-terminus or C-terminus of WIPI3-Δloop to stabilize the complex or facilitate crystallization. After extensive trials, the crystals of WIPI3-Δloop with N-terminal fusion of the WIR-peptide were obtained for structural determination (Fig. 1a).

### Structure of the WIPI3-Δloop/WIR-peptide complex

The complex structure was determined by the molecular replacement method and refined to 2.2 Å (Supplementary Table 1). The WIR-peptide of ATG2A was resolved (from 1378 to 1399) based on its electron density map (Fig. 1a–c). Consistent with the stoichiometry of 1:1 in the ITC assay, a single WIR-peptide binds to WIPI3-Δloop (Fig. 1b and Supplementary Fig. 1d). In the crystal packing, the C-terminus of the WIR-peptide from one molecule is linked to the N-terminus of WIPI3-Δloop from another molecule (Supplementary Fig. 2), which accidentally promotes the packing of neighboring molecules for crystallization. This may explain why WIPI3-Δloop only with N-terminal fusion of the WIR-peptide could be crystallized. Moreover, based on the crystal packing analysis, the fusion of the WIR-peptide to WIPI3-Δloop did not induce any distortion of the packing between them and had little impact on the complex structure (Supplementary Fig. 2).

In the complex structure, WIPI3-Δloop adopts a seven-bladed β-propeller fold and each blade contains four anti-parallel β-strands (from A to D) (Fig. 1b, d). The β-propeller resembles a disk-like structure with a narrower top and a wider bottom from the side view and the WIR-peptide forms a linear structure and tightly entwines around the β-propeller from the rim to bottom (Fig. 1e). From overall structure, the WIR-peptide spans across blades 1–3 and binds to multiple inter-blade sites embedded in the β-propeller (Fig. 1d, e). The two deleted loops in WIPI3 are far away from these inter-blade sites and would not impact on the complex formation, consistent with the similar binding affinities of WIR-peptide for WIPI3 and WIPI3-Δloop (Supplementary Figs. 3a and  1c, d). Moreover, WIPI3 contains two conserved phosphoinositide-binding sites in blades 5–6 (characterized with the signature “LRRG” motif), which do not overlap but are opposite to the peptide-binding sites in blades 1–3 (Fig. 1f). Thus, WIPI proteins tend to recognize phosphoinositides and the WIR-peptide simultaneously, consistent with their versatile functions as autophagic phosphoinositide effectors.

### Interaction interface between WIPI3 and the WIR-peptide

**Fig. 2:**
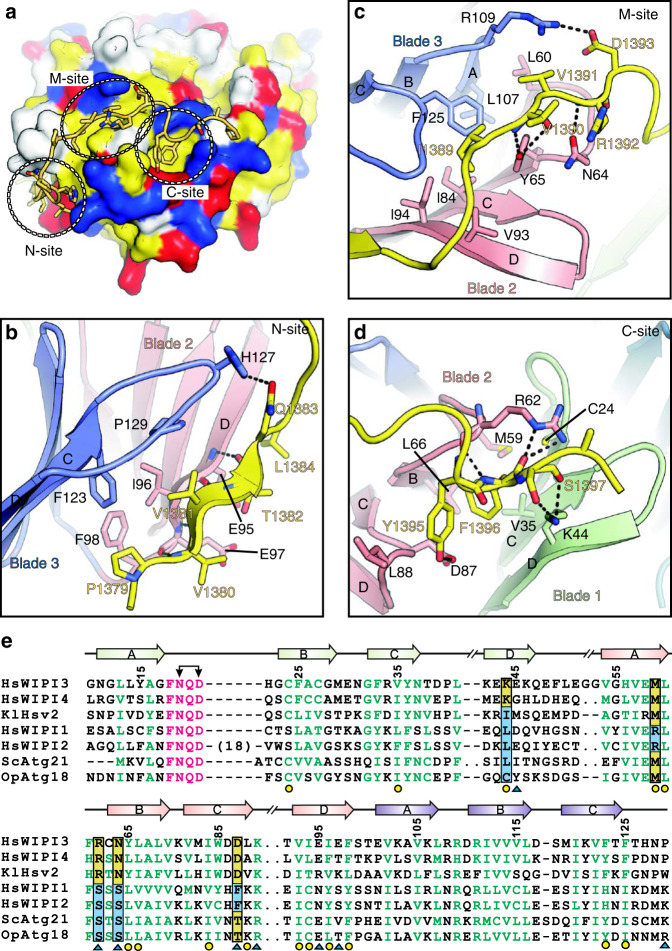
Interaction interface between WIPI3 and the WIR-peptide. **a** A combined surface and stick representation showing the interaction interface between WIPI3 and the WIR-peptide. The WIR-peptide is in the stick representation (colored in yellow) and WIPI3 is in the surface representation (the hydrophobic, positively charged, negatively charged residues, and the rest of the residues are colored in yellow, blue, red, and white, respectively). The three interaction sites (N-site, M-site, and C-site) are highlighted by dashed circles. **b**–**d** A combined ribbon-and-stick model illustrates the interaction interface between WIPI3 and the WIR-peptide. WIPI3 and the WIR-peptide are colored as that in Fig. 1b and the sidechains of the residues involved in the interface packing are shown as sticks. **e** Structure-based sequence alignment of human WIPI proteins, yeast Hsv2, Atg21, and Atg18. The identical and highly conserved residues are colored in magenta and green, respectively. The secondary structures and residues numbers of WIPI3 are marked at the top. The hydrophobic and polar residues responsible for the WIR-peptide interaction are marked with yellow dots and blue triangles respectively, at the bottom. The key residues of C-site that are different in WIPI proteins are indicated by black boxes. The conserved asparagine and aspartic acid in PROPPIN proteins are indicated by black arrows.

The interaction interface between the WIR-peptide and WIPI3 can be divided into three sites (namely N-site, M-site, and C-site, from the N-terminus to C-terminus of the WIR-peptide) (Fig. 2a). In N-site, the N-terminal part of the WIR-peptide forms a short β-strand to augment the β-strand D of blade 2 with specific hydrogen bonds between their backbones; P1379 and V1381 from the WIR-peptide make hydrophobic contacts with F98 and I96 from blade 2 and F123 and P129 from blade 3; the sidechain of Q1383 forms an additional hydrogen bond with the sidechain of H127 (Fig. 2b, e). In M-site, I1389 and V1391 from the middle segment of the WIR-peptide insert into the hydrophobic pocket formed by I94, V93, I84, Y65, and L60 from blade 2 and L107 and F125 from blade 3; the backbones of V1390 and R1392 from the WIR-peptide form hydrogen bonds with the sidechains of Y65 and N64 from blade 2, respectively; D1393 from the WIR-peptide forms an electrostatic interaction with R109 from blade 3 (Fig. 2c, e). In C-site, the C-terminal aromatic tail of the WIR-peptide packs into a tailored pocket formed between blades 1 and 2, i.e., Y1395 and F1396 from the WIR-peptide make hydrophobic contacts with L88, L66, and M59 from blade 2 and C24 and V35 from blade 1; the sidechain of Y1395 from the WIR-peptide forms a hydrogen bond with the sidechain of D87 from blade 2; the backbones of Y1395, F1396, and S1397 from the WIR-peptide form an extensive hydrogen-bonding network with the backbone and sidechain of R62 from blade 2 and the sidechain of K44 from blade 1, and the sidechain of K44 also seems to line up with the sidechain of F1396, together with R62, to lock F1396 in this pocket (Fig. 2d, e). Taken together, the extensive hydrophobic, hydrogen-bonding, and electrostatic interactions in these three sites contribute to the formation of the WIPI3/WIR-peptide complex.

### Three peptide-binding sites in WIPI proteins

**Fig. 3:**
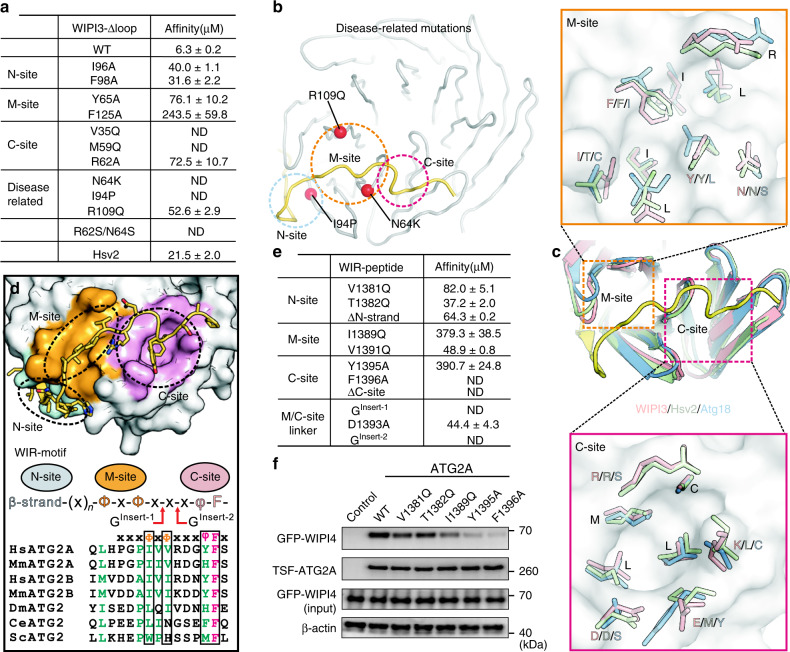
Three peptide-binding sites in WIPI3 and the WIR-motif for WIPI proteins. **a** A summary of the binding affinities between WIR-peptide and WIPI3-Δloop (and its mutants) determined by the ITC assay. ND not detectable. **b** A combined cartoon and sphere representation showing the disease-related point mutations in human WIPI3 (R109Q) and WIPI4 (N64K and I94P, the corresponding position in WIPI3). **c** Structural comparison of the WIPI3/WIR-peptide complex, Hsv2 (PDB code: 4EXV), and Atg18 (PDB code: 5LTG). The key residues of M-site and C-site in WIPI3 and the corresponding residues in Hsv2 and Atg18 are highlighted and shown by sticks. **d** The WIR-motif for binding to WIPI proteins. In the sequence alignment of ATG2 family proteins, the identical and highly conserved residues are colored in magenta and green, respectively, and the consensus residues are indicated by black boxes. **e** A summary of the binding affinities between WIPI3-Δloop and the WIR-peptide mutants determined by the ITC assay. ND not detectable. **f** Strep-pull-down analysis of the interactions between WIPI4 and full-length ATG2A (and its mutants). TSF-ATG2A Tandem-Strep-Flag-tagged ATG2A. The Strep-pull-down experiment was repeated twice independently with similar results. Source data are provided as a Source Data file.

To evaluate the essential roles of these three sites in WIPI3 for recognizing the WIR-peptide, we made point mutations in each site and performed the ITC assay to measure the binding affinities between the mutants and WIR-peptide. All the point mutations were introduced in WIPI3-Δloop because we hardly obtained sufficient amount of full-length WIPI3 mutants for the binding assay (Supplementary Fig. 4). As expected, point mutations in N-site (I96A and F98A), M-site (Y65A and F125A), or C-site (V35Q, M59Q, and R62A) severely impaired the interaction between WIPI3-Δloop and the WIR-peptide (Figs. 2b–d, 3a), supporting that the three peptide-binding sites in WIPI3 are all essential for recognizing the WIR-peptide. Notably, mutations in M-site and C-site seem to have more impact on the WIR-peptide binding (Fig. 3a), suggesting that M-site and C-site are probably primary peptide-binding sites and N-site is an auxiliary one.

To further analyze the three peptide-binding sites in detail, we compared the structure of the WIPI3/WIR-peptide complex with that of apo-WIPI3. The two structures could be well superimposed with each other (with the RMSD of ~0.43 Å for the backbone atoms), but upon binding of the WIR-peptide, the A–B loop of blade 2 in C-site underwent local conformational changes (Supplementary Fig. 3b). Specifically, in apo-WIPI3, the sidechain of F61 points into the peptide-binding pocket and the sidechain of R62 faces outside to form a hydrogen bond with the sidechain of Q20 (Supplementary Fig. 3c), while in the WIPI3/WIR-peptide complex, the backbone of F1396 from the WIR-peptide takes over this hydrogen bond and forms a hydrogen-bonding network with the sidechain and backbone of R62 (Fig. 2d), which would likely lead to the flipping of the sidechain of neighboring F61 and the further change in the backbone position of the A–B loop of blade 2 (Supplementary Fig. 3c). Thus, among the three peptide-binding sites, both N-site and M-site are largely preformed, but C-site needs to undergo conformational reorganizations in the A–B loop of blade 2 (i.e., repositioning of F61 and R62) to open up the Y1395-F1396-binding pocket, resembling a gatekeeper for WIPI3 to recognize the WIR-peptide.

Previous studies demonstrated that the two residues in the A–B loop of blade 1 (i.e., N15 and D17 in WIPI4, N19 and D21 in WIPI3) are essential for WIPI4 to interact with ATG2A^7^ (Fig. 2e and Supplementary Fig. 5a). We further made the mutations of these two residues in WIPI3. Consistent with previous studies, the mutations (N19A/D21A) abolished the binding of the WIR-peptide to WIPI3 (Supplementary Fig. 5b), confirming that the A–B loop of blade 1 is also essential for the binding. Based on the WIPI3/WIR-peptide complex structure, the A–B loop of blade 1 does not interact with the WIR-peptide directly but is neighboring to the A–B loop of blade 2 in C-site (Fig. 1b). More importantly, the sidechain of N19 from the A–B loop of blade 1 forms a hydrogen bond with the backbone of F61 from the A–B loop of blade 2 (Supplementary Fig. 5a). As aforementioned in structural comparison, the A–B loop of blade 2 needs to undergo local conformational changes to accommodate the bulky aromatic tail of the WIR-peptide (Supplementary Fig. 3c). Thus, it is possible that the A–B loop of blade 1 can facilitate the conformational changes of the A–B loop of blade 2 and stabilizes the formation of the target-binding pocket in C-site for recognizing the WIR-peptide.

Mutations in WIPI proteins (WIPI3 and WIPI4) have been found to be linked with human diseases^12,26,27^. Among these mutations, three point mutations (N64K, I94P, and R109Q) are located in peptide-binding sites (Fig. 3b). To evaluate the effects of these diseased-related mutations on peptide binding, we further generated the N64K, I94P, and R109Q mutations in WIPI3-Δloop and checked the interactions of three mutants with the WIR-peptide. As expected, all the mutations severely disrupted the binding to the WIR-peptide (Fig. 3a), supporting the potential correlation between the WIR-peptide binding and diseases.

### Potential peptide-binding sites in PROPPIN β-propellers

WIPI3 and WIPI4 were both reported to interact with ATG2 proteins^20^. Consistent with this, the critical residues responsible for the formation of three peptide-binding sites in WIPI3 are highly conserved in WIPI4 (Fig. 2b–e), suggesting that WIPI4 binds to ATG2A with a similar mode. Supporting this hypothesis, the ATG2A/B-binding region in WIPI4 was previously narrowed down to blades 1–3 that match the three peptide-binding sites in WIPI3^20,21^. In contrast, the key residues in C-site (K44, M59, R62, and D87) for recognizing the aromatic tail of the WIR-peptide are not conserved in WIPI1 and WIP2 (Fig. 2d, e), implying that C-site may be missing from WIPI1 and WIPI2 or this potential peptide-binding site in WIPI1 and WIPI2 would not recognize the aromatic tail. Consistent with this assumption, WIPI1 and WIPI2 cannot bind to ATG2 but interact with a set of different proteins^9,20^. Instead of the peptide sequence containing aromatic residues, WIPI2 was reported to recognize a short stretch of peptide from ATG16L enriched with negatively charged residues^9^. On the other hand, we further made the point mutations of R62 and N64 in WIPI3 (that are two serines in WIPI1-2) to somewhat mimic the potential target-binding sites in WIPI1-2 (Fig. 2e). As expected, the mutations of R62 and N64 to two serines severely impaired the interaction between WIPI3 and the WIR-peptide from ATG2A (Fig. 3a), suggesting that the deviation of the potential target-binding sites in WIPI1-2 may cause the lack of the binding to ATG2 proteins. Thus, the differences of the potential peptide-binding sites between WIPI proteins (WIPI1-2 versus WIPI3-4) seem to reflect their intrinsic capabilities to recruit different proteins for controlling the different stages of autophagosome formation. Nevertheless, the mechanism underlying the peptide-recognition by WIPI1 and WIPI2 still needs further investigations.

To investigate the potential peptide-binding sites in other PROPPINs, we then compared the WIPI3/WIR-peptide complex structure with the available Hsv2 and Atg18 structures. Intriguingly, the three peptide-binding sites in WIPI3 could be well-aligned with the corresponding inter-blade pockets in Hsv2 and Atg18 (Fig. 3c), indicating that these inter-blade pockets are also the potential peptide-binding sites in other PROPPINs. Consistent with this analysis, in the Atg18/Atg2 complex, the Atg2-binding region in Atg18 was found to be primarily located in blade 2 that is the central core of three peptide-binding sites^19^. More intriguingly, most of the essential residues in the three peptide-binding sites of WIPI3 are highly conserved in Hsv2, especially in M-site and C-site (Fig. 3c), indicating that Hsv2 may be able to recognize the WIR-like-peptide. Expectedly, the ITC assay showed that Hsv2 can indeed interact with the WIR-peptide of ATG2A albeit with a weaker affinity (with the Kd of 21.5 ± 2.0 μM) (Fig. 3a and Supplementary Fig. 4), thus supporting that the similar peptide-recognition mode may happen in Hsv2 and the peptide-binding sites found in WIPI3 could be extended to other PROPPINs.

### The WIR-motif for binding to WIPI proteins

The WIR-peptide in the complex structure can be divided into the N-terminal part, middle segment, and C-terminal tail, corresponding to the three sites in the interaction interface. The N-terminal part tends to form a short β-strand, the middle segment contains two separated hydrophobic residues, and the C-terminal tail is featured with two consecutive aromatic residues (Fig. 3d). We made point mutations in each part of the WIR-peptide (V1381Q, I1389Q, V1391Q, Y1395A, and F1396A) to evaluate the essential roles of these residues for binding to WIPI3. As expected, all the mutations severely impaired the binding to WIPI3-Δloop (Fig. 3e and Supplementary Fig. 6). Consistent with mutational studies of WIPI3 (Fig. 3a), mutations in the middle segment and C-terminal tail of the WIR-peptide have more impact on the binding (Fig. 3e), supporting the primary roles of M-site and C-site and the auxiliary function of N-site.

Among the three parts of the WIR-peptide, the N-terminal β-strand seems to be dynamic as indicated by the relatively higher value of B-factor (Supplementary Fig. 7a, b). To evaluate this β-strand for the complex formation, we made the point mutation of each residue of “^1380^VVT^1382^” to alanine or glutamine (in addition to the V1381Q mutation). Interestingly, the mutations of these residues had different impacts on the binding to WIPI3. Specifically, the V1380A (rather than V1380Q) mutation affected the binding, while the T1382Q (rather than T1382A) mutation impacted the binding (Fig. 3e and Supplementary Fig. 7c, d). In contrast, both the V1381A and V1381Q mutations decreased the binding more severely than the mutations of V1380 and T1382 (Fig. 3e and Supplementary Fig. 7c, d), consistent with that V1381 is located in the interaction interface and contributes more to the binding to WIPI3 (Fig. 2b). Thus, the mutations of the three residues all somewhat impaired the binding, confirming the essential role of this N-terminal β-strand for the complex formation. On the other hand, we further made the truncation of the N-terminal β-strand (ΔN-strand) or the C-terminal aromatic tail (ΔC-site) of the WIR-peptide. The ΔN-strand mutant still could bind to WIPI3 with a moderate affinity (with the Kd of 64.3 ± 0.2 μM), but the ΔC-site mutant showed no binding to WIPI3 (Fig. 3e), consistent with the assumption that C-site is the primary peptide-binding site and N-site is the auxiliary one.

In the interaction interface between WIPI3 and the WIR-peptide, the linker between M-site and C-site is composed of three residues (R1392, D1393, and G1394) that form a sharp turn structure (Fig. 2a), which is maintained by the hydrogen-bonding interaction between the backbone of R1392 and the sidechain of N64 and the electrostatic interaction between D1393 and R109 (Fig. 2c). In addition to the N64K and R109Q mutations in WIPI3, we further made the D1393A mutation in the WIR-peptide. As expected, all the mutations disrupted the binding between WIPI3 and the WIR-peptide (Fig. 3a, e). To further check the role of this M/C-site linker in the WIR-peptide for binding to WIPI3, we inserted a glycine between R1392 and D1393 (to disrupt the packing of the linker to WIPI3) or between D1393 and G1394 (without affecting the packing of R1392 and D1393 to WIPI3) (Figs. 2c and 3d), and to our surprise, both the two glycine-insertion mutations abolished the binding to WIPI3, similar to the severe effect caused by the mutation of F1396 (Fig. 3e). One possible explanation is that the glycine-insertion would not only extend the linker length but also increase the intrinsic flexibility of the aromatic tail, which would lead to the mismatch between the aromatic tail and C-site. Taken together, the M/C-site linker is also essential for the WIR-peptide to bind to WIPI3.

Based on all these mutational studies, the WIR-motif for binding to WIPI3 and WIPI4 can be defined as the signature sequence of “β-strand-(X)_n_-Φ-X-Φ-X-X-X-Ψ-F” (Φ is a hydrophobic residue and Ψ is an aromatic residue), which is highly conserved in ATG2 family members (Fig. 3d). In the WIR-motif, the N-terminal β-strand contains no specified residues, and we then searched out a number of new potential WIPI-binding proteins based on the middle hydrophobic segment and C-terminal aromatic tail (Fig. 3d and Supplementary Table 2). Among these potential binding partners, some of them (e.g., ATG13, FIP200, and VPS15) have been indicated to function in the control of autophagic process. More specifically, recent studies demonstrated that WIPI3 can interact with FIP200 to form a stable complex and integrates the LKB-AMPK-TSC signaling for controlling autophagy^7^. Based on this study, the potential WIR-motif in FIP200 is likely to be capable of mediating the interaction between WIPI3 and FIP200, albeit further confirmation is needed. On the other hand, some of these potential binding partners are involved in controlling membrane trafficking (that has been suggested to contribute to autophagosome biogenesis), suggesting that WIPI proteins may also play an essential role in the linkage of membrane trafficking to autophagosome formation. Nevertheless, the potential binding partners in Supplementary Table 2 were searched out purely based on the primary sequence analysis, whether these proteins interact with and function together with WIPI3/4 awaits further investigations.

### The WIR-motif in ATG2A is essential for autophagic process

**Fig. 4:**
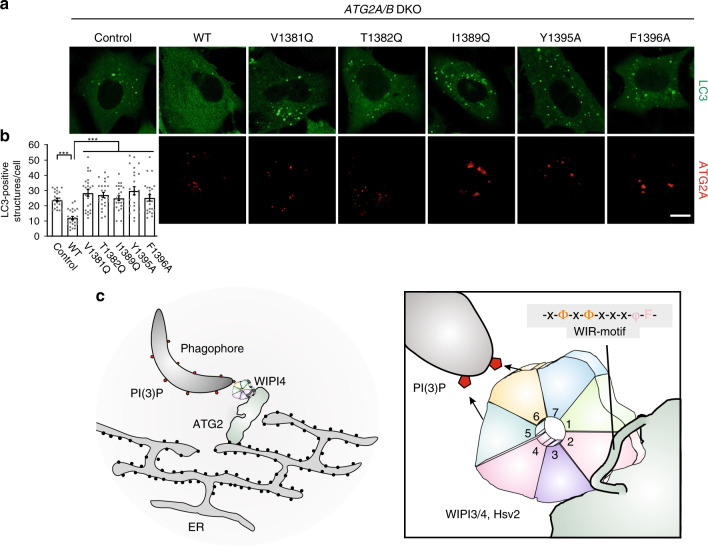
The WIR-motif in ATG2A is essential for the ATG2A-mediated autophagic process. **a** Fluorescence images of NRK cells with double knock out (DKO) of *ATG2A*/*ATG2B* and stable expression of GFP-LC3. Wild-type ATG2A could rescue the depletion defect but the mutants could not. Scale bar: 10 μm. **b** Quantification of the abnormal LC3-positive structures shown in **a**. The number of LC3-positive structures per cell was quantified (*n* = 25). Each bar represents the mean value ± SEM. ***p < 0.001, unpaired, two-tailed Student’s *t* test. Source data are provided as a Source Data file. **c** A schematic working model for the formation of the WIPI4/ATG2 complex at the ER-phagophore junction and the WIR-motif-recognition by WIPI β-propellers. Briefly, WIPI proteins specifically recognize the linear WIR-motif that resembles a rope to entwine around WIPI β-propellers. WIPI β-propellers bind to phosphoinositides and effector proteins simultaneously and the WIPI4/ATG2 complex bridges phagophores with ER membranes.

Finally, we evaluated the functional importance of the WIR-motif in ATG2A using its in vivo cognate partner WIPI4. We first made point mutations in the WIR-motif of full-length ATG2A and checked the binding between these ATG2A mutants and WIPI4 by the previously established Strep-pull down assay^20^. As expected, mutations in each part of the WIR-motif in ATG2A (V1381Q, T1382Q, I1389Q, Y1395A, and F1396A) decreased the binding to WIPI4 (Fig. 3f), supporting that the WIR-motif is essential for ATG2A to interact with WIPI4. Moreover, the formation of the ATG2A/WIPI4 complex was proposed to be important for the expansion and maturation of autophagosome and depletions of ATG2 proteins in mammalian cells led to the accumulation of immature phagophores^21,28^. To evaluate the essential role of the WIR-motif in ATG2A for its cellular functions, we further used the normal rat kidney epithelial (NRK) cells with depletions of both ATG2A and ATG2B and stable expression of GFP-tagged LC3 and also checked the autophagic flux with the presence of bafilomycin A1 (an inhibitor of autophagosome–lysosome fusion). Consistent with recent studies^29^, depletions of ATG2 proteins resulted in the abnormal accumulation of large LC3-positive structures under nutrient-rich condition (Fig. 4a). No obvious increase of these LC3-positive structures was observed in the presence of bafilomycin A1 (Supplementary Fig. 8). As expected, transfection of wild-type ATG2A could rescue the depletion defect with significant decreasing of large LC3-positive clusters (Fig. 4a, b), and LC3-positive clusters were markedly re-accumulated after adding bafilomycin A1 (Supplementary Fig. 8). In contrast, transfection of all the WIR-motif mutants (V1381Q, T1382Q, I1389Q, Y1395A, and F1396A) could not rescue the depletion defect (Fig. 4a, b). Although the cellular level of LC3-positive structures was also increased after adding bafilomycin A1, the increments of LC3-positive clusters (with transfection of the ATG2A mutants) were significantly lower than that upon transfection of wild-type ATG2A (Supplementary Fig. 8). Thus, the conserved WIR-motif in ATG2A mediates the binding to WIPI4 and is essential for the ATG2A-mediated autophagic process.

A series of recent studies demonstrated that ATG2 proteins are essential for phagophore expansion and autophagosome maturation and are able to interact with WIPI4 to form a stable WIPI4/ATG2 complex^20,28,29^. Moreover, both WIPI4 and ATG2A can be localized at the junctions between phagophores and ER membranes (where nascent autophagosomes are generated)^7^. Structural studies of the WIPI4/ATG2A complex demonstrated that this complex adopts a rod-shaped structure with two opposite tips tethering two different membranes (i.e., PI(3)P-containing and PI(3)P-free membranes)^21^. Meanwhile, ATG2 proteins were also found to contain the lipid-transfer capacity to mediate the lipid-transfer between two membranes^29,30^. In this study, we found that the WIR-motif in ATG2A is responsible for binding to WIPI4 and mutations of the WIR-motif disrupted the interaction between WPI4 and ATG2A and impaired the ATG2A-mediated autophagic process (Figs. 3f and 4a). Thus, in the proposed working model (Fig. 4c), the WIPI4/ATG2A complex would be able to bridge phagophores with ER membranes and position two different membranes for the direct transfer of lipids from ER membranes to phagophores for autophagosome formation. Disruptions of the interaction between WIPI4 and ATG2A would dissociate the WIPI4/ATG2A complex and break the bridges between phagophores and ER membranes, which would lead to the accumulation of immature phagophores, consistent with functional studies of ATG2 proteins.

In summary, this work reveals the multi-site-mediated peptide-recognition mechanism and the spatial arrangement of the peptide-binding and phosphoinositide-binding sites of WIPI β-propellers, which enables them to bind to phosphoinositides and effector proteins simultaneously by different blades for regulating autophagosome formation, e.g., the formation of the WIPI4/ATG2 complex at the ER-phagophore junction for autophagosome biogenesis (Fig. 4c).

## Methods

### Protein expression and purification

DNA sequences encoding human WIPI3, WIPI3-Δloop, and two ATG2A fragments (1358–1404 and 1374–1404) were each cloned into a modified pET32a vector. The generation of the fusion construct of WIPI3-Δloop with ATG2A(1374–1404) and the mutations in WIPI3 and the ATG2A fragment were performed by using the standard PCR-based mutagenesis method and confirmed by DNA sequencing. All the primers used in the study were listed in Supplementary Table 3. Recombinant proteins were expressed in *Escherichia coli* BL21(DE3) (Invitrogen, C6000-03) host cells at 16 °C. The GB1-His_6_-tagged fusion proteins were purified by Ni^2+^-Sepharose 6 Fast Flow (GE healthcare) affinity chromatography using the wash buffer (50 mM Tris-HCl, pH 8.0, 500 mM NaCl, 25 mM imidazole) and elution buffer (50 mM Tris-HCl, pH 8.0, 500 mM NaCl, 500 mM imidazole). The eluted proteins were further purified by size-exclusion chromatography (Superdex-200 26/60, GE healthcare). For WIPI3 proteins, after cleavage of the tag, the resulting proteins were purified by another step of size-exclusion chromatography with the buffer containing 50 mM Tris-HCl, pH 8.0, 100 mM NaCl, 1 mM EDTA, 1 mM DTT. For ATG2A fragments, recombinant proteins were purified in the same buffer without cleavage of the tag.

### Isothermal titration calorimetry (ITC) assay

ITC assay was performed using a MicroCalorimeter ITC200 (GE Healthcare Life Sciences, USA) at 25 °C. Prior to the experiment, the protein samples were dialyzed in the buffer containing 50 mM Tris-HCl, pH 8.0, 100 mM NaCl, 1 mM EDTA, and 1 mM DTT. WIPI3 proteins were put in the sample cell and GB1-tagged ATG2A fragments were in the syringe of the instrument. In each experiment, the ATG2A fragment was sequentially injected into the stirred calorimeter cell initially containing the WIPI3 protein sample with the injection sequence of 20 × 2 µl at 2-min intervals. The heat of dilution obtained by the titration of the ATG2A fragment into the buffer was subtracted. The integrated, corrected, and concentration-normalized peak areas of the raw data were finally fitted with a model of one binding site using ORIGIN 7.0 (OriginLab).

### Crystallization, data collection, and structural determination

Purified WIPI3 proteins were concentrated to ~20 mg/ml in the buffer containing 50 mM Tris-HCl pH 8.0, 100 mM NaCl, 1 mM EDTA, and 50 mM DTT for crystallization. Crystals of the WIPI3-Δloop/WIR-peptide complex (the WIR-peptide-WIPI3-Δloop fusion protein) were grown in 0.1 M Tris-HCl, pH 8.5, and 20% (v/v) Ethanol and were obtained using the vapor diffusion method (sitting drop) at 16 °C. Before being flash-frozen in liquid nitrogen, crystals were cryo-protected in their mother liquor supplemented with 16% (v/v) ethylene glycol. Diffraction data were collected at the beamline BL19U at the Shanghai Synchrotron Radiation Facility (SSRF) using with a wavelength of 0.979 Å at 100K^31^, and were processed and scaled using HKL2000^32^. The structure were determined by the molecular replacement method with the structure of WIPI3 (PDB code: 6IYY [10.2210/pdb6iyy/pdb]) as searching models using PHASER^33^. The WIR-peptide was manually modeled into the structure according to the 2Fo-Fc and Fo-Fc electron density maps using COOT^34^. The structures were further fitted and refined with PHENIX^35^. The structure figures were prepared with the program PyMOL. The statistics for data collection and structural refinement were summarized in Supplementary Table 1.

### Strep-pull down assay

GFP-tagged WIPI4 and Tandem-Strep-Flag (TSF)-tagged ATG2A (wild-type or mutants) plasmids were co-transfected into HEK293T cells (ATCC, CRL-3216) using Lipofectamine 3000 transfection reagent (Thermo Fisher Scientific). Cells were collected 48 h after transfection. Cell pellets were lysed in lysis buffer containing 50 mM Tris-HCl, pH 8.0, 150 mM NaCl, 10 mM DTT, 1 mM EDTA, 1 × Protease inhibitor cocktail, 1 mM PMSF, and 1% (v/v) NP-40. Lysates were centrifuged at 20,000 *g* for 30 min at 4 °C to separate soluble fractions and cell debris. Supernatants were applied to Strep-Tactin Sepharose beads (IBA) and incubated for 1 h at 4 °C. The beads and non-bound proteins were separated by centrifugation at 1000 *g* at 4 °C. After washing the beads three times with a wash buffer composed of 50 mM Tris-HCl, pH 8.0, 150 mM NaCl, 10 mM DTT, 1 mM EDTA, and 0.2% (v/v) NP-40, the bound proteins were eluted with the elution buffer containing 50 mM Tris-HCl, pH 8.0, 150 mM NaCl, 10 mM DTT, and 5 mM desthiobiotin (IBA). The eluted products were analyzed by SDS-PAGE. GFP-tagged WIPI4 and TSF-tagged ATG2A were detected by western blot using the anti-GFP (Proteintech, 66002-1-Ig, 1:5000 dilution) and anti-Flag (Sigma-Aldrich, F1804, 1:5000 dilution) primary antibodies, respectively, and the goat anti-mouse secondary antibody (Abcam, ab97023, 1:5000 dilution). Overall 1% of the input was loaded onto SDS-PAGE as the loading control and detected by the anti-beta-actin antibody (Abcam, ab8226, 1:5000 dilution).

### NRK cell culture and imaging

NRK cells with double knock out of *ATG2A/ATG2B* and stably transfected with GFP-LC3 were generous gifts from L.Y., Tsinghua University. DNA sequences encoding full-length ATG2A and its various mutants were cloned into the pCAG-Tandem-Strep-Flag vector. Cells were cultured in DMEM (GIBCO) supplemented with 10% (v/v) fetal bovine serum, 100 U/ml penicillin and 0.1 mg/ml streptomycin and were grown at 37 °C in a humidified atmosphere containing 5% (v/v) CO_2_. Cells were transfected with wild-type ATG2A and various mutants by the electroporation apparatus (Bio-Rad) according to the manufacturer’s instructions. After 48 h, cells were washed three times by PBS and fixed by 4% (w/v) paraformaldehyde. For bafilomycin A1 treatment, cells were cultured in the complete medium containing 10 nM bafilomycin A1 for 2 h before being fixed. ATG2A and its various mutants were detected by immunofluorescence with the anti-Flag primary antibody (Sigma-Aldrich, F1804, 1:300 dilution) and dylight 594 secondary antibody (EarthOx, E032410-01, 1:500 dilution). Fluorescence images were obtained on an inverted confocal microscope (LSM 880, Carl Zeiss) equipped with a 100 × oil-immersion objective lens (NA = 1.30). Confocal settings used for image capture were held constant in comparison experiments. All the fluorescence images were processed and analyzed using the ZEN imaging software (Carl Zeiss). Microsoft Excel and GraphPad Prism were used for statistic analysis.

### Reporting summary

Further information on experimental design is available in the Nature Research Reporting Summary linked to this paper.

## Supplementary information

Supplementary Information

Reporting summary

## Data Availability

The atomic coordinate of the WIPI3-Δloop/WIR-peptide complex has been deposited in the Protein Data Bank with the accession code 6KLR [10.2210/pdb6klr/pdb]. The atomic coordinates used for molecular replacement or structural comparison were download from the Protein Data Bank (6IYY [10.2210/pdb6iyy/pdb], 4EXV [10.2210/pdb4exv/pdb], and 5LTG [10.2210/pdb5ltg/pdb]). The source data underlying Figs. 3f and 4b and Supplementary Fig. 8b are provided as a Source Data file. Other data that support the findings of this study are available from the corresponding author upon reasonable request.

## Source Data

Source Data
