# Supplementary material for: Multi-site-mediated entwining of the linear WIR-motif around WIPI β-propellers for autophagy
Source: Nat Commun. 2020 Jun 1;11:2702. doi: 10.1038/s41467-020-16523-y (PMC7264293; doi:10.1038/s41467-020-16523-y)
Supplement: Supplementary file 1 — Supplementary Information [file 41467_2020_16523_MOESM1_ESM.pdf]

Supplementary Information

Multi-site-mediated entwining of the linear WIR-motif around WIPI β-propellers for autophagy

Jinqi Ren, Ruobing Liang, Wenjuan Wang et al.

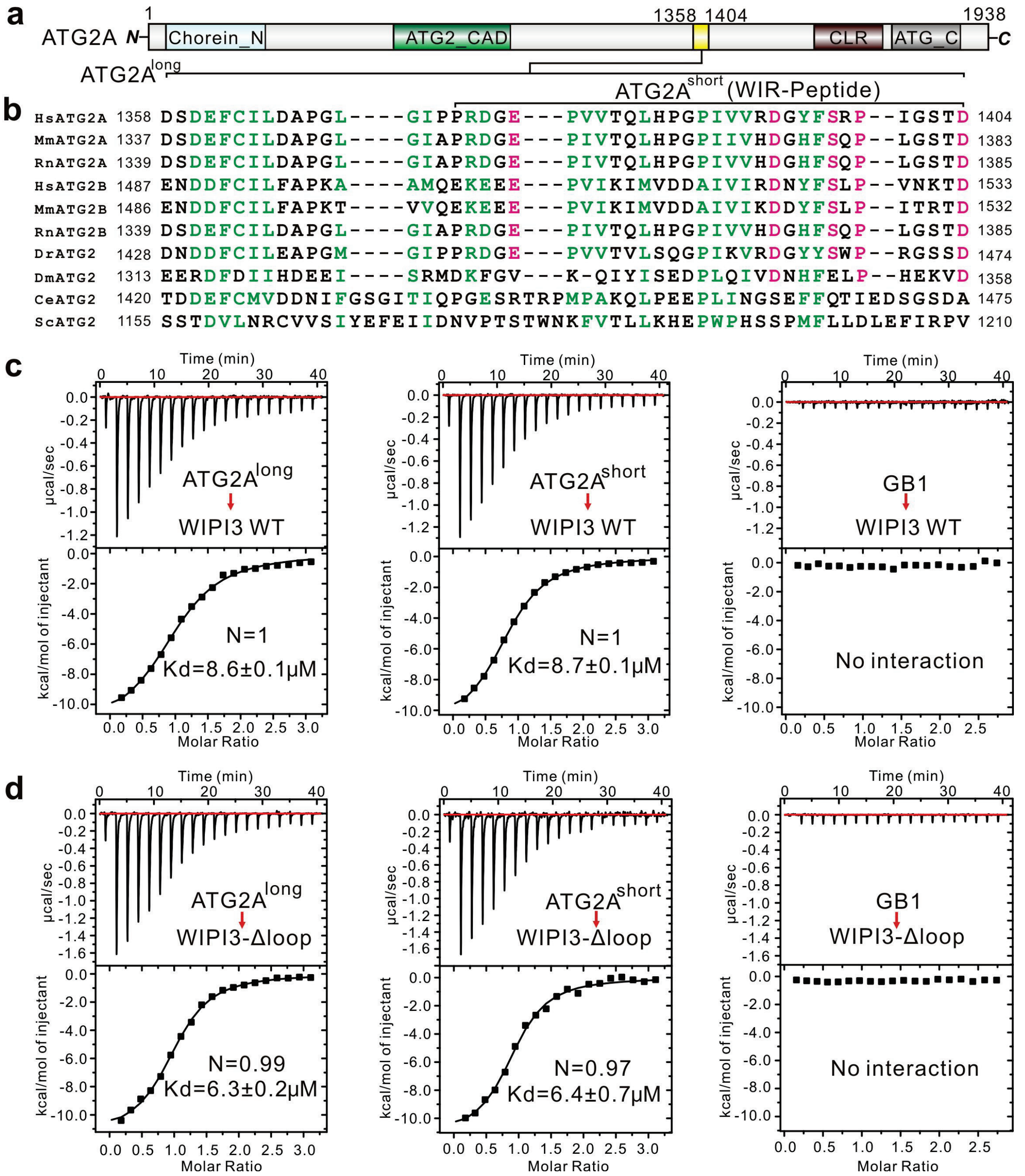

Supplementary Fig. 1 Biochemical characterization of the interactions between WIPI3 and the two fragments of ATG2A. (a) Domain organization of ATG2A. ATG2A contains the N-terminal Chorein_N domain, the ATG2_CAD domain in the middle and the C-terminal tandem CLR-ATG_C domain. The WIPI-interacting region of ATG2A has been narrowed down to residues 1358-1404 and is colored in yellow. **(b)** Sequence alignment of ATG2 family proteins from different species. The identical residues are colored in magenta and the highly conserved residues are colored in green. The WIR-peptide is the short fragment of ATG2A from residues 1374 to 1404. Hs, *Homo sapiens*; Mm, *Musmusculus*; Rn, *Rattusnorregicus*; Dn, *Deniorerio*; Ce, *Caenorhabditiselegans*; Dm, *Drosophila melanogaster*; Sc, *Saccharomyces cerevisiae*. **(c-d)** The binding affinities between the

different fragments of ATG2A (ATG2A^{long} and ATG2A^{short}) and WIPI3 (c) and that between the different fragments of ATG2A and WIPI3-Δloop (d) determined by ITC experiments. GB1 was used as the control. The binding affinities are indicated in each panel.

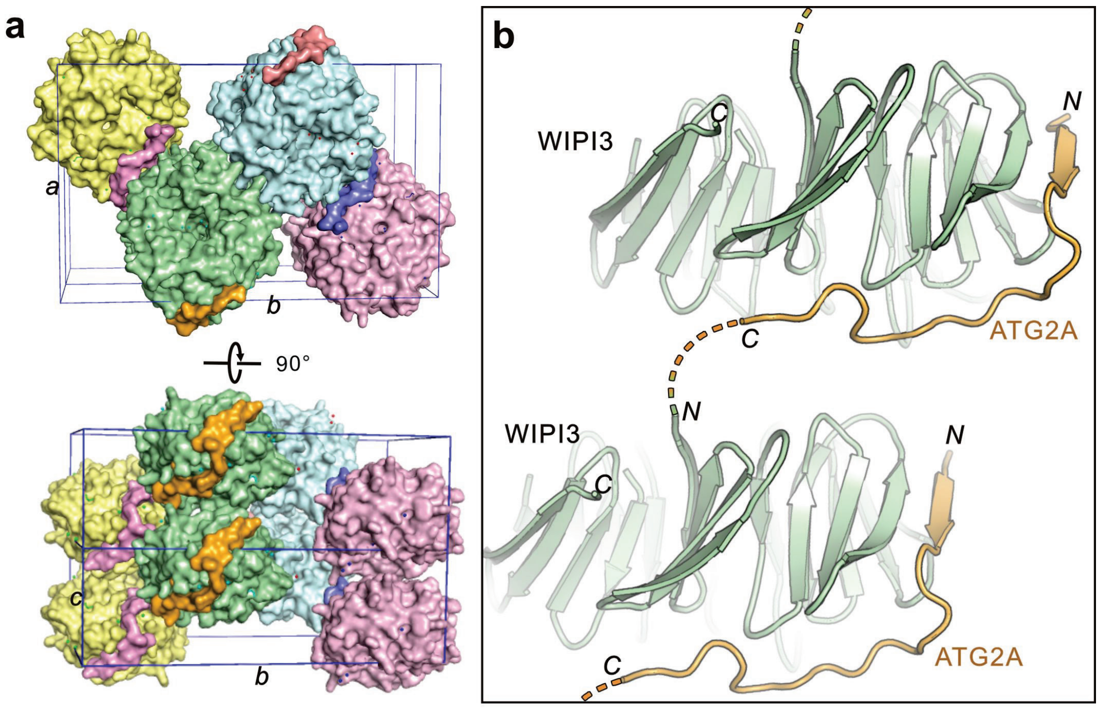

Supplementary Fig. 2 Crystal packing analysis of the WIPI3-Δloop/WIR-peptide complex. (a) An orthogonal view of packing molecules in two unit cells from different angles. Two adjacent unit cells along the a axis were depicted. **(b)** A close-up view of two neighboring molecules in crystal packing. The flexible linker (indicated by a dashed line) that connects the WIR-peptide with WIPI3-Δloop is untraceable in the structure. In two neighboring molecules, the C-terminus of the WIR-peptide from one molecule is linked to the N-terminus of WIPI3-Δloop from the other, which would promote the packing between them for crystallization.

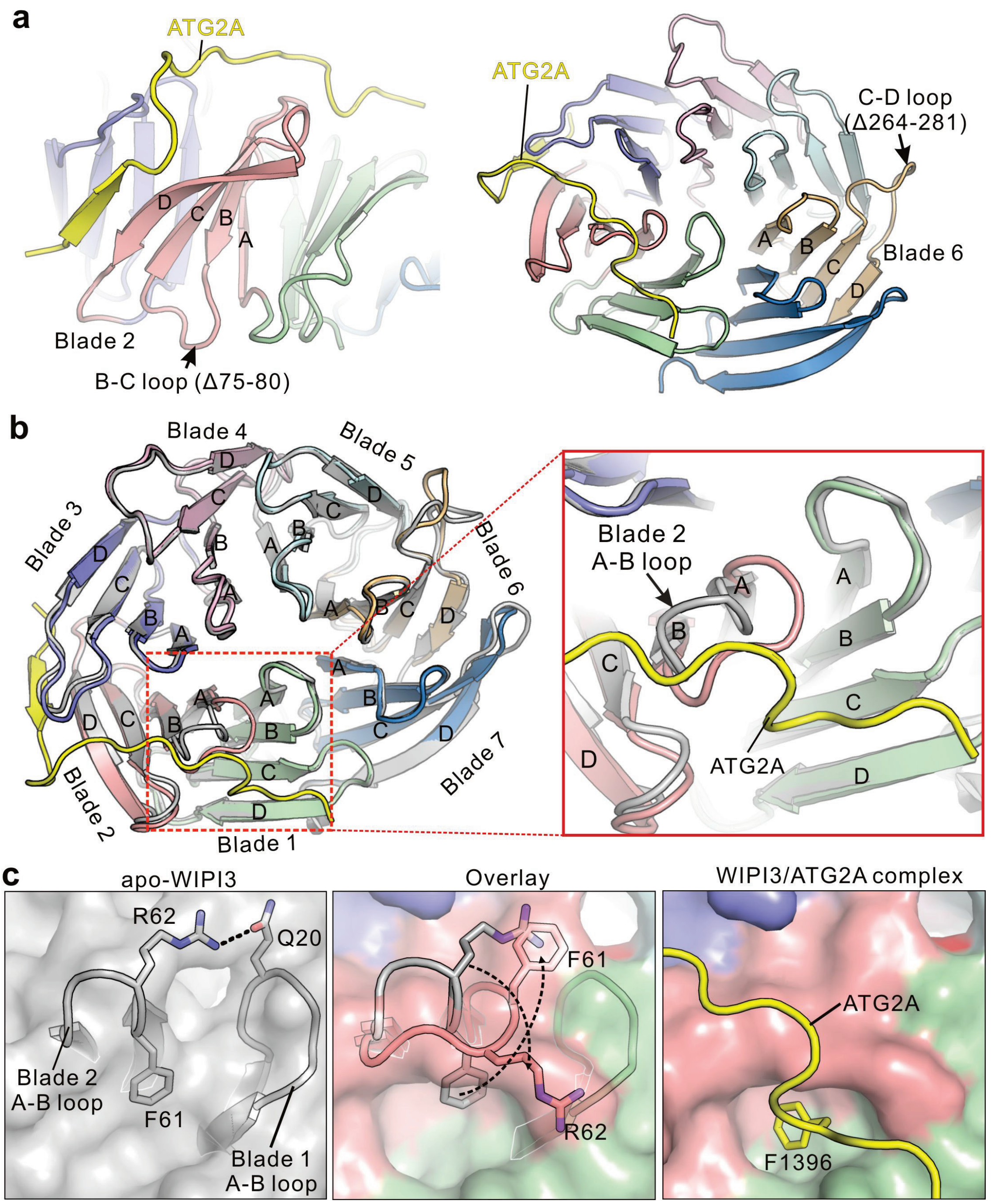

Supplementary Fig. 3 Structure of the WIPI3/WIR-peptide complex and comparison with apo-WIPI3. (a) Deletion of two flexible loops in WIPI3 has little impact on the complex formation. The B-C loop in blade 2 and the C-D loop in blade 6 were removed for crystallization (indicated by arrows). Both the two loops are far away from the peptide-binding sites and would not impact on the complex formation. **(b)** Superimposition of the structures of apo-WIPI3 (colored in grey, PDB code: 6IYY) and the WIPI3/WIR-peptide complex. The WIPI3/WIR-peptide complex aligns well with apo-WIPI3 except for certain local conformational changes in the A-B loop of blade 2 (as shown in a close-up view). **(c)** Conformational changes of the A-B loop in blade 2 upon binding of the WIR-peptide.

Compared with apo-WIPI3, the sidechains of F61 and R62 in the WIPI3/ATG2A complex flip oppositely to accommodate the bulky aromatic residue F1396 from the WIR-peptide.

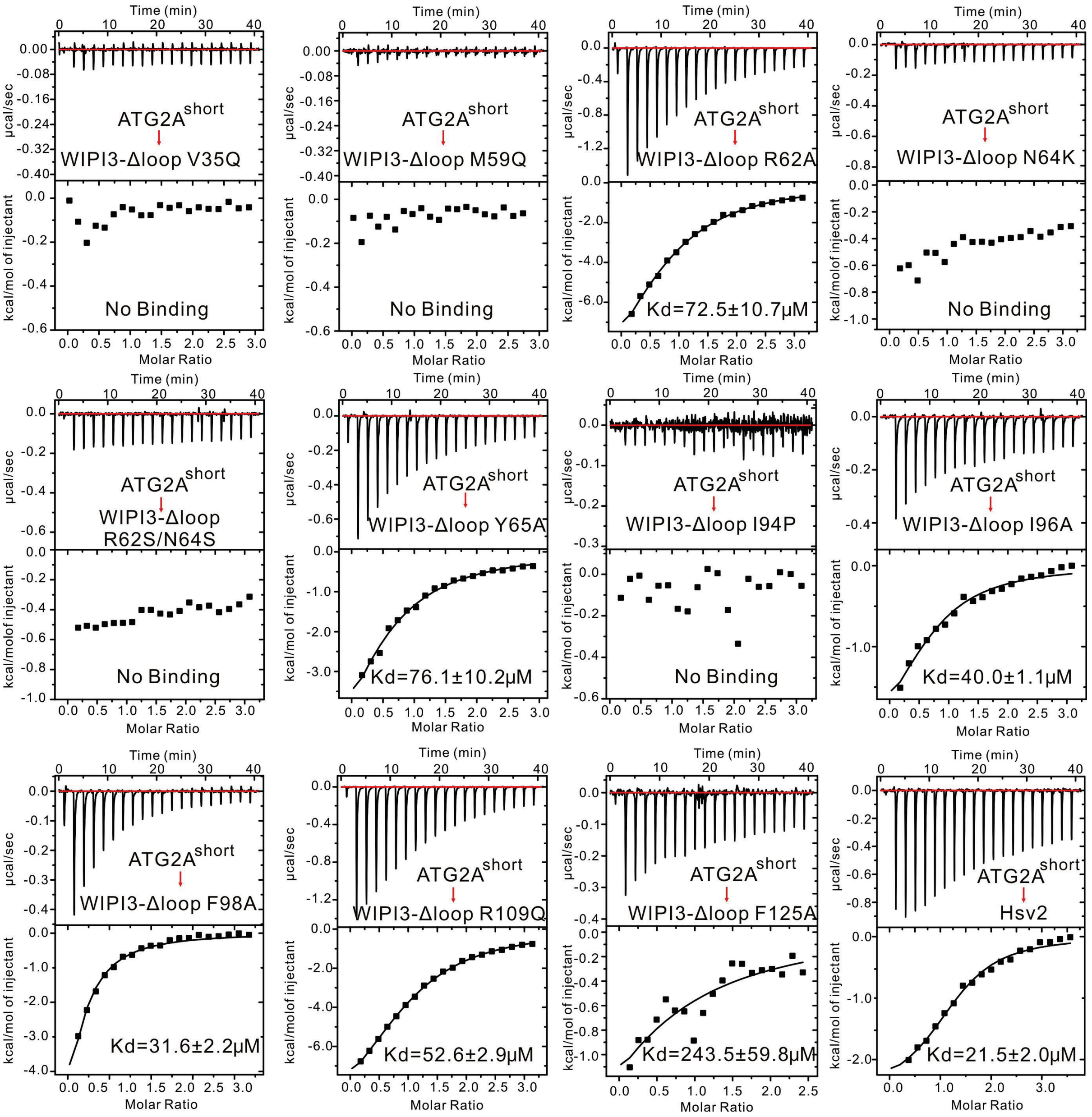

Supplementary Fig. 4 Biochemical characterization of the bindings between the WIR-peptide from ATG2A and various WIPI3-Δloop mutants. The binding affinities between the WIR-peptide and various WIPI3-Δloop mutants determined by ITC experiments. The interaction of Hsv2 with the WIR-peptide from ATG2A is also summarized in this figure. The binding affinities are indicated in each panel.

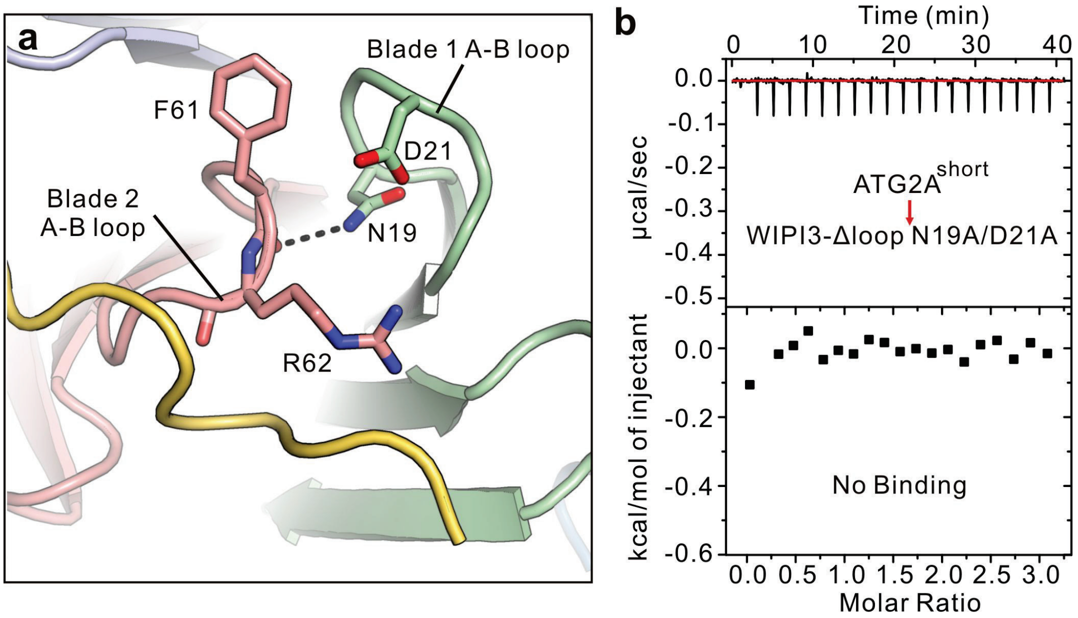

Supplementary Fig. 5 Structural analysis of the A-B loop of blade 1. (a) The A-B loop of blade 1 is neighboring to the A-B loop of blade 2 in C-site and the sidechain of N19 from the A-B loop of blade 1 forms a hydrogen bond with the backbone of F61 from the A-B loop of blade 2, which would stabilize the formation of this target-binding pocket. **(b)** The binding affinity between the WIR-peptide and the WIPI3-Δloop N19A/D21A mutant determined by ITC experiments. The N19A/D21A mutation abolished the interaction between the WIR-peptide and WIPI3-Δloop.

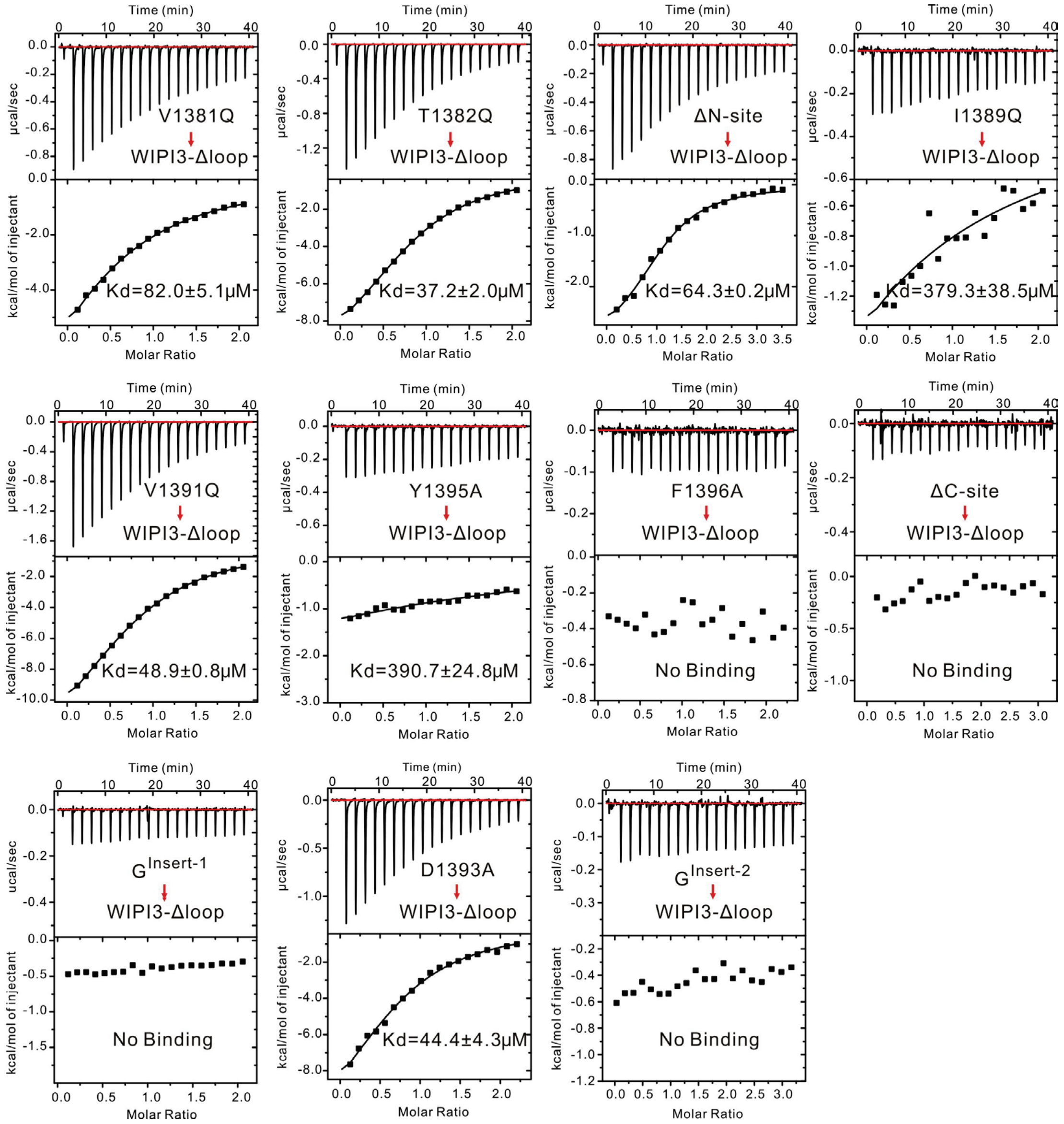

Supplementary Fig. 6 Biochemical characterization of the bindings between WIPI3-Δloop and various WIR-peptide mutants. The binding affinities between WIPI3-Δloop and various WIR-peptide mutants determined by ITC experiments. The binding affinities are indicated in each panel.

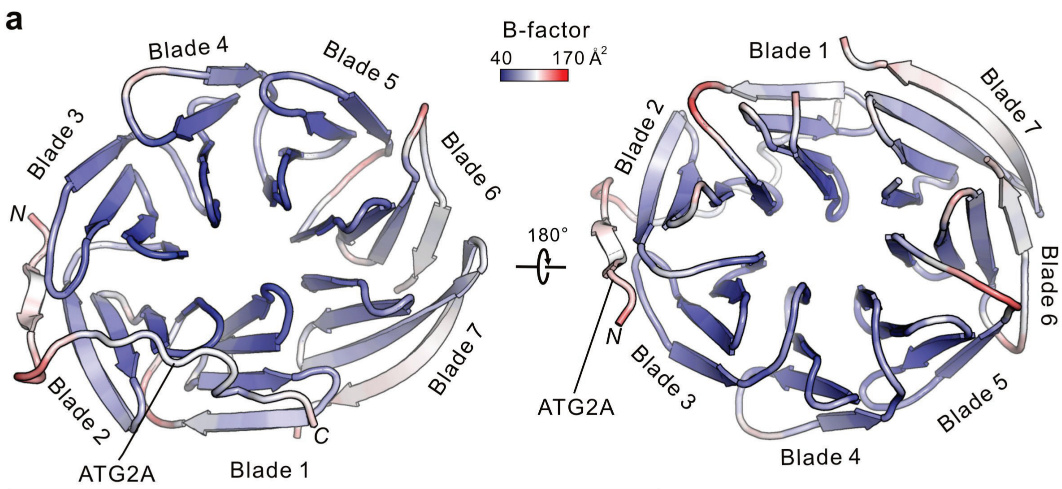

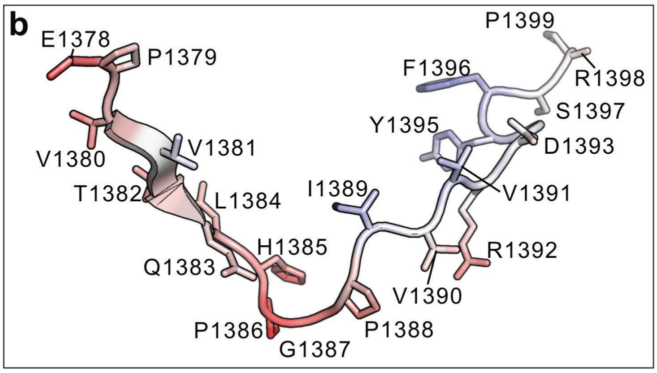

c

WIR-Peptide	Affinity(μM)
V1380A	36.9±4.0
V1380Q	14.1±3.7
V1381A	75.9±4.0
T1382A	14.2±2.3

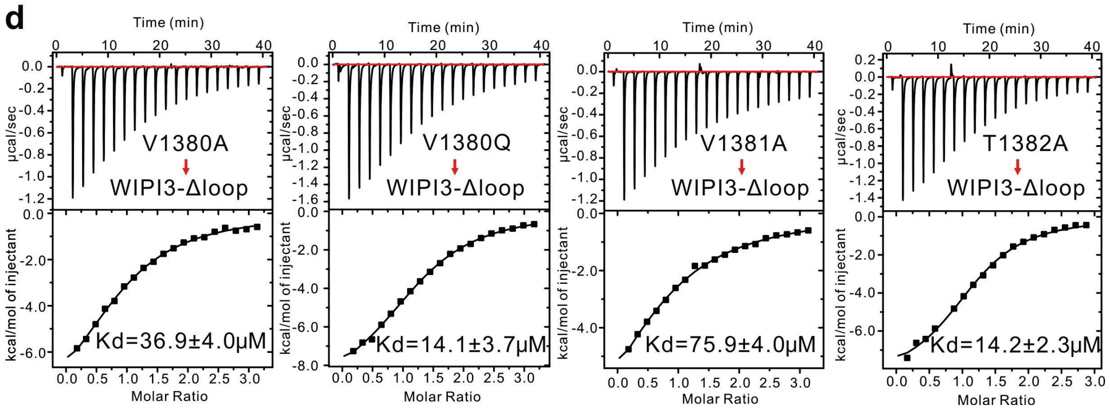

Supplementary Fig. 7 Biochemical characterization of the N-terminal β-strand of the WIR-peptide. (a) The structural model of the WIPI3/WIR-peptide complex colored with the B-factor (according to the value of the B-factor, colored from blue through white to red). **(b)** A close-up view of the WIR-peptide. The N-terminal β-strand of the WIR-peptide possesses the relatively higher value of the B-factor than the other parts of the WIR-peptide. **(c)** A summary of the binding affinities between WIPI3-Δloop and the WIR-peptide with mutations in the N-terminal β-strand. **(d)** The ITC assay of the bindings between WIPI3-Δloop and the WIR-peptide mutants. The binding affinities are indicated in each panel.

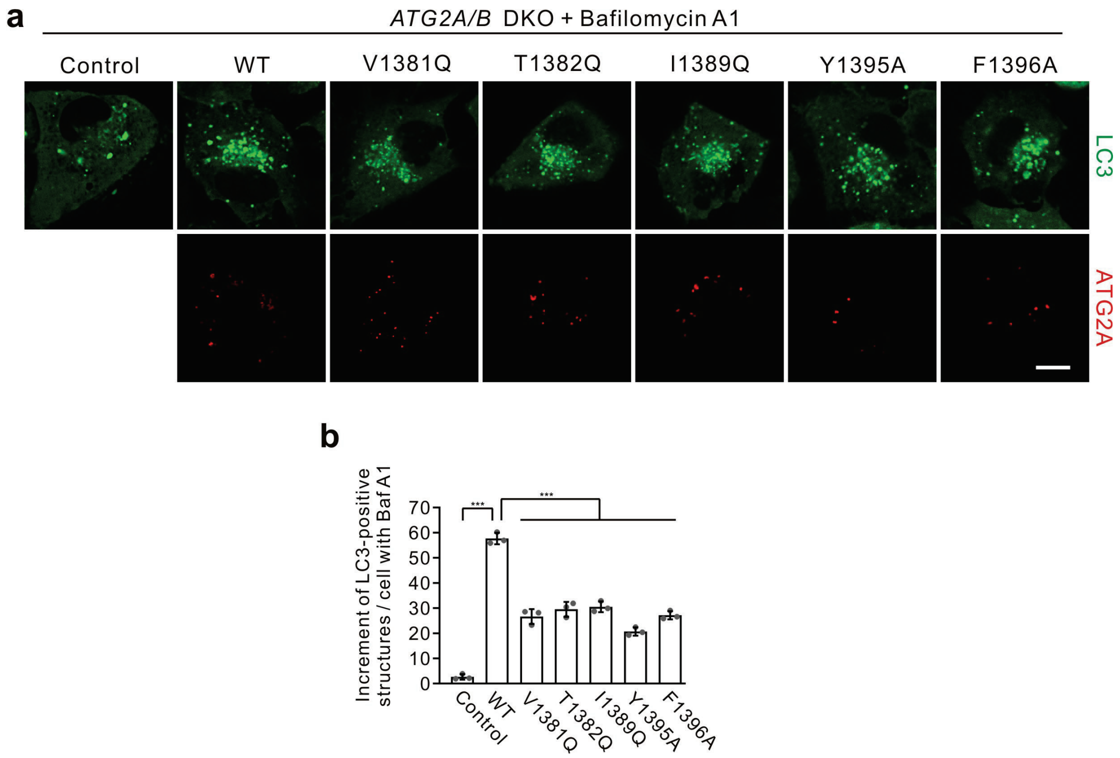

Supplementary Fig. 8 Analysis of the autophagic flux in the presence of bafilomycin A1. (a) Fluorescence images of NRK cells (with double knock out (DKO) of *ATG2A*/*ATG2B* and stable expression of GFP-LC3) after the treatment of bafilomycin A1. After adding bafilomycin A1, no obvious increase of LC3-positive structures was observed in the control, but the LC3-positive clusters were increased in the cells upon transfection of wild-type ATG2A or the ATG2A mutants. Scale bar: 10 µm. **(b)** Quantification of the increment of LC3-positive structures with bafilomycin A1 treatment. The increment of LC3-positive structures per cell was quantified with three sets of data. In each dataset, the number of LC3-positive structures per cell (without or with bafilomycin A1 treatment) was first averaged for more than 8 cells and then the difference between their average numbers of LC3-positive structures was used for quantification ($n = 3$). Each bar represents the mean value ±SD. ***$p < 0.001$, unpaired, two-tailed Student's t-test. Source data are provided as a Source Data file.

Supplementary Table 1 Data collection and structural refinement statistics

	The WIPI3-Δloop/WIR-peptide complex
**Data collection**	
Space group	$P2_12_12$
Cell dimensions	
a, b, c (Å)	75.12, 121.06, 37.77
α, β, γ (°)	90, 90, 90
Resolution (Å)	50.0-2.2 (2.28-2.20) *
R_{merge}	0.097 (0.423)
$I / \sigma(I)$	20.7 (3.2)
Completeness (%)	99.3 (96.2)
Redundancy	11.6 (7.9)
**Refinement**	
Resolution (Å)	24.5-2.2
No. reflections	17638
R_{work} / R_{free}	21.3 / 25.5
No. atoms	
Protein	2467
Water	18
B-factors	
Protein	85.4
Water	67.5
R.m.s. deviations	
Bond lengths (Å)	0.007
Bond angles (°)	0.91

The number of crystals used for structure determination and refinement is one.

*Values in parentheses are for highest-resolution shell.

Supplementary Table 2 A list of putative WIR-motif-containing proteins in human proteome

Protein name(Uniprot Accession ID)	Start	Sequences	End	Function
WIR-motif		ϕXϕXXXφF		
Autophagy-related protein 2A (Q2TAZ0)	1389	IVVRDGYF	1396	Autophagy
Autophagy-related protein 2B (Q96BY7)	1518	IVIRDNYF	1525	Autophagy
Synaptotagmin-16 SYT16 (Q17RD7)	59	IQIQETYF	66	Exocytosis
LRATD1 (Q96KN4)	29	LRVGVAYF	36	Cell motility
Alstrom syndrome protein 1 (Q8TCU4)	2745	VGVFNSHF	2752	Endosomal transport
Synaptotagmin-like protein 5 (Q8TDW5)	211	LDLDGQHF	218	Exocytosis
Autophagy-related protein 13 (O75143)	207	MGIIIDHF	214	Autophagy
Phosphoinositide 3-kinase regulatory subunit 4 VPS15 (Q99570)	13	ILSVESYF	20	Autophagy
RB1-inducible coiled-coil protein 1FIP200 (Q8TDY2)	803	VVAQDSHF	810	Autophagy
Deleted in lung and esophageal cancer protein 1 (Q9Y238)	1127	FSLKFEYF	1134	Tumor suppressor
Uncharacterized protein C2orf42 (Q9NWW7)	388	FHIPQSFF	395	Unknown
Vacuolar protein sorting-associated protein 11 homolog (Q9H270)	894	FSVIADYF	901	Protein trafficking
Metastasis-associated protein MTA3 (Q9BTC8)	467	FFLHTTYF	475	Transcription repression
Myoferlin (Q9NZM1)	891	IKLKREFF	898	Endocytic recycling
Spermatogenesis-associated protein 45 (Q9UM82)	43	LRVQKRHF	50	Necroptosis
Macrophage receptor MARCO (Q9UEW3)	74	LRVLEMYF	81	Pattern recognition
Lysosomal-trafficking regulator(Q99698)	2351	IKLLDAYF	2358	Endosomal transport
Tyrosine-protein kinase JAK3 (P52333)	342	VALVDGYF	349	Signal transduction
Leucine-rich repeat serine/threonine-protein kinase 2 (Q5S007)	1590	LQLSDLYF	1597	Retrograde trafficking
NACHT, LRR and PYD domains-containing protein 3 (Q96P20)	1003	LGLSEMYF	1010	Inflammation
E3 ubiquitin-protein ligase UBR4 (Q5T4S7)	2284	VTFPIDFF	2291	Protein degradation

Nanos homolog 2 (Q8WY41)	5	FDMWKDYF	12	Translational repressor
Bloom syndrome protein (P54132)	1318	IPVSSHYF	1325	DNA helicase
Unconventional myosin-XV MYO15A (Q9UKN7)	3012	LEFAQKYF	3019	Intracellular movements
Pygopus homolog 1 (Q9Y3Y4)	168	VNMPNQHF	175	Signal transduction
UBX domain-containing protein 6 (Q9BZV1)	285	FELPGDFF	292	Macroautophagy
HEAT repeat-containing protein 5B (Q9P2D3)	2063	IKLKTSFF	2070	Endocytosis
Inositol 1,4,5-trisphosphate receptor type 2 (Q14571)	1483	MNIVSGFF	1490	Intracellular Ca^{2+} release
Grainyhead-like protein 3 homolog (Q8TE85)	457	LFIPNVHF	464	Transcription factor
Kinesin-like protein KIF1A (Q12756)	910	ISFDDQHF	918	Intracellular movements

*Both protein name and Uniprot accession ID are provided. The position and sequence of the putative WIR-motif are also shown.

Supplementary Table 3 Primers used in this study

Name	Sequence(5'-3')
WIPI3	
WIPI3-1-F	CTGTTCCAGGGCCCCGGATCCATGAACCTCCTG
WIPI3-344-R	GTGGTGGTGGTGGTGCTCGAGTCACAGCTTGTCATC
WIPI3-8-F	CTGTTCCAGGGCCCCGGATCCCCTCACGGCAAC
Del75-80-F	TTTAGTTGGTGGTGGAAAAAAGAAAGTAATGATCTGGGATGACC
Del75-80-R	GGTCATCCCAGATCATTACTTTCTTTTTTCCACCACCAACTAAA
Del264-281-F	TGCATATTTTTGCAGCTGAAGATCCAAAATCCAAGTGGAGTTTC
Del264-281-R	GAAACTCCACTTGGATTTTGGATCTTCAGCTGCAAAAATATGCA
V35Q-F	GTGGGATGGAAAATGGATTCCGACAGTATAACACTGATCCACTAAAAGA
V35Q-R	TCTTTTAGTGGATCAGTGTTATACTGTCGGAATCCATTTTCCATCCCAC
K44A-F	GTCTATAACACTGATCCACTAAAAGAAGCAGAGAAACAAGAATTTCTAGAAGGA
K44A-R	TCCTTCTAGAAATTCTTGTTTCTCTGCTTCTTTTAGTGGATCAGTGTTATAGAC
M59Q-F	GGAGGAGTTGGCCATGTTGAACAGTTATTTCGCTGCAACTATTT
M59Q-R	AAATAGTTGCAGCGAAATAACTGTTCAACATGGCCAACTCCTCC
R62A-F	GGAGTTGGCCATGTTGAAATGTTATTTGCCTGCAACTATTTAGC
R62A-R	GCTAAATAGTTGCAGGCAAATAACATTTCAACATGGCCAACTCC
N64A-F	CATGTTGAAATGTTATTTCGCTGCGCCTATTTAGCTTTAGTTGGTGGTGG
N64A-R	CCACCACCAACTAAAGCTAAATAGGCGCAGCGAAATAACATTTCAACATG
Y65A-F	GTTGAAATGTTATTTCGCTGCAACGCTTTAGCTTTAGTTGGTGGTGGAAA
Y65A-R	TTTCCACCACCAACTAAAGCTAAAGCGTTGCAGCGAAATAACATTTCAAC
F98A-F	ACCTGAAGAAGAAGACTGTTATTGAAATAGAAGCTTCTACAGAAGTCAAGG
F98A-R	CCTTGACTTCTGTAGAAGCTTCTATTTCAATAACAGTCTTCTTCTTCAGGT
F125A-F	CCATGATTAAGGTGTTCACAGCCACACACAATCCCCATCAGT
F125A-R	ACTGATGGGGATTGTGTGTGGCTGTGAACACCTTAATCATGG
I96A-F	ATGACCTGAAGAAGAAGACTGTTATTGAAGCAGAATTTTCTACAGAAGTCAAG
I96A-R	CTTGACTTCTGTAGAAAATTCTGCTTCAATAACAGTCTTCTTCTTCAGGTCAT
F61A-F	GGAGGAGTTGGCCATGTTGAAATGTTAGCTCGCTGCAACTATT
F61A-R	AATAGTTGCAGCGAGCTAACATTTCAACATGGCCAACTCCTCC
N64K-F	TTGAAATGTTATTTCGCTGCAAGTATTTAGCTTTAGTTGGTGGTG
N64K-R	CACCACCAACTAAAGCTAAATACTTGCAGCGAAATAACATTTCAA
I94P-F	CTGGGATGACCTGAAGAAGAAGACTGTTCCTGAAATAGAATTTTCTACAGAA
I94P-R	TTCTGTAGAAAATTCTATTTCAGGAACAGTCTTCTTCTTCAGGTCATCCCAG
R109Q-F	GCAGTCAAGCTGCGGCAAGATAGAATTGTGGTG
R109Q-R	CACCACAATTCTATCTTGCCGCAGCTTGACTGC
N19A/D21A-F	CTCTACGCCGGCTTCGCCCAGGCCCACGGATGCTTTGC
N19A/D21A-R	GCAAAGCATCCGTGGGCCTGGGCGAAGCCGGCGTAGAG
R62S/N64S-F	GGCCATGTTGAAATGTTATTTAGCTGCAGCTATTTAGCTTTAGTTGGTGG
R62S/N64S-R	CCACCAACTAAAGCTAAATAGCTGCAGCTAAATAACATTTCAACATGGCC
ATG2A	
V1381A-F	GATGGGGAGCCTGTGGCTACACAGCTGCATCC
V1381A-R	GGATGCAGCTGTGTAGCCACAGGCTCCCCATC
T1382A-F	GATGGGGAGCCTGTGGTGGCACAACTGCATCC

T1382A-R	GGATGCAGTTGTGCCACCACAGGCTCCCCATC
Ginsert-1-F	GGCCCCATCGTTGTGAGGGGTGACGGTTACTTCTCACGGCCGATC
Ginsert-1-R	GATCGGCCGTGAGAAGTAACCGTCACCCCTCACAACGATGGGGCC
ATG2A-1358-F	CTGTTCCAGGGCCCCGGATCCGACAGTGATGAG
ATG2A-1387-F	CTGTTCCAGGGCCCCGGATCCGGCCCCATCGTTG
ATG2A-1404-R	GTGGTGGTGGTGGTGCTCGAGTCAGTCCGTGCTGCCG
ATG2A-1394-R	GTGGTGGTGGTGGTGCTCGAGTCAACCGTCCCTCAC
ATG2A-1374-F	CTGTTCCAGGGCCCCGGATCCCCCCCGAGATGG
V1380Q-F	CGAGATGGGGAGCCTCAGGTGACACAGCTG
V1380Q-R	CAGCTGTGTCACCTGAGGCTCCCCATCTCG
V1381Q-F	GATGGGGAGCCTGTGCAGACACAGCTGCATCC
V1381Q-R	GGATGCAGCTGTGTCTGCACAGGCTCCCCATC
T1382Q-F	GGGGAGCCTGTGGTGCAGCAGCTGCATCCCGGC
T1382Q-R	GCCGGGATGCAGCTGCTGCACCACAGGCTCCCC
I1389Q-F	CTGCATCCCGGCCCCCAGGTTGTGAGGGACGGT
I1389Q-R	ACCGTCCCTCACAACCTGGGGGCCGGGATGCAG
V1391Q-F	CATCCCGGCCCCATCGTTCAGAGGGACGGT
V1391Q-R	ACCGTCCCTCTGAACGATGGGGCCGGGATG
D1393A-F	CCATCGTTGTGAGGGCCGGTTACTTCTCACG
D1393A-R	CGTGAGAAGTAACCGGCCCTCACAACGATGG
Y1395A-F	CGTTGTGAGGGACGGTGCCTTCTCACGGCCGATC
Y1395A-R	GATCGGCCGTGAGAAGGCACCGTCCCTCACAACG
F1396A-F	GTGAGGGACGGTTACGCCTCACGGCCGATCGG
F1396A-R	CCGATCGGCCGTGAGGCGTAACCGTCCCTCAC
pmC-1-F	GCTCAAGCTTCGAATTCTGCAGTCGACATGTCACGATGGCTGTGGCCATGG
pmC-1938-R	GCTCACCATGGTGGCGACCGGTGGATCCCGGTCTTGGGCACTGTCCGAGCGCC
V1380A-F	GATGGGGAGCCTGCGGTGACACAGCTG
V1380A-R	CAGCTGTGTCACCGCAGGCTCCCCATC
Ginsert-2-F	GGCCCCATCGTTGTGAGGGACGGTGGTTACTTCTCACGGCCGATC
Ginsert-2-R	GATCGGCCGTGAGAAGTAACCACCGTCCCTCACAACGATGGGGCC
