# Supplementary material for: Multi-site-mediated entwining of the linear WIR-motif around WIPI β-propellers for autophagy
Source: Nat Commun. 2020 Jun 1;11:2702. doi: 10.1038/s41467-020-16523-y (PMC7264293; doi:10.1038/s41467-020-16523-y)
Supplement: Supplementary file 2 — Reporting summary [file 41467_2020_16523_MOESM2_ESM.pdf]

nature research

Corresponding author(s): Wei Feng
Last updated by author(s): Apr 30, 2020

Reporting Summary

Nature Research wishes to improve the reproducibility of the work that we publish. This form provides structure for consistency and transparency in reporting. For further information on Nature Research policies, see Authors & Referees and the Editorial Policy Checklist .

Statistics

For all statistical analyses, confirm that the following items are present in the figure legend, table legend, main text, or Methods section.

n/a	Confirmed	
☐	☒	The exact sample size (*n*) for each experimental group/condition, given as a discrete number and unit of measurement
☐	☒	A statement on whether measurements were taken from distinct samples or whether the same sample was measured repeatedly
☐	☒	The statistical test(s) used AND whether they are one- or two-sided<br>*Only common tests should be described solely by name; describe more complex techniques in the Methods section.*
☒	☐	A description of all covariates tested
☒	☐	A description of any assumptions or corrections, such as tests of normality and adjustment for multiple comparisons
☐	☒	A full description of the statistical parameters including central tendency (e.g. means) or other basic estimates (e.g. regression coefficient) AND variation (e.g. standard deviation) or associated estimates of uncertainty (e.g. confidence intervals)
☐	☒	For null hypothesis testing, the test statistic (e.g. *F*, *t*, *r*) with confidence intervals, effect sizes, degrees of freedom and *P* value noted<br>*Give P values as exact values whenever suitable.*
☒	☐	For Bayesian analysis, information on the choice of priors and Markov chain Monte Carlo settings
☒	☐	For hierarchical and complex designs, identification of the appropriate level for tests and full reporting of outcomes
☒	☐	Estimates of effect sizes (e.g. Cohen's *d*, Pearson's *r*), indicating how they were calculated

Our web collection on statistics for biologists contains articles on many of the points above.

Software and code

Policy information about availability of computer code

Data collection	ITC data collection: iTC200. Fluorescence images collection: Carl Zeiss imaging software ZEN2.3 SP1.
Data analysis	X-ray data process: HKL2000 version 720. Structural determination: PHASER version 2.8.3. Structural model building: COOT version 0.8.9.2. Structural refinement: PHENIX version 1.17. ITC data analysis and fitting: Origin version 7.0. Figure preparation: PyMol version 1.7.0.0. Statistic analysis: Microsoft Excel version 2010, GraphPad Prism version 8.0.2.

For manuscripts utilizing custom algorithms or software that are central to the research but not yet described in published literature, software must be made available to editors/reviewers. We strongly encourage code deposition in a community repository (e.g. GitHub). See the Nature Research guidelines for submitting code & software for further information.

Data

Policy information about availability of data

All manuscripts must include a data availability statement. This statement should provide the following information, where applicable:

- Accession codes, unique identifiers, or web links for publicly available datasets
- A list of figures that have associated raw data
- A description of any restrictions on data availability

The structural factors and atomic coordinate reported in this paper have been deposited in the Protein Data Bank with the accession code 6KLR [http://dx.doi.org/10.2210/pdb6klr/pdb]. Other protein coordinates used for molecular replacement (PDB: 6IYY [http://dx.doi.org/10.2210/pdb6iyy/pdb]) or structural comparison (PDB: 4EXV [http://dx.doi.org/10.2210/pdb4exv/pdb] and PDB: 5LTG [http://dx.doi.org/10.2210/pdb5ltg/pdb]) are all publicly accessible.
The source data underlying Figs. 3f and 4b and Supplementary Fig. 8b are provided as a Source Data file.
Other data are available from the corresponding author upon reasonable request.

Field-specific reporting

Please select the one below that is the best fit for your research. If you are not sure, read the appropriate sections before making your selection.

- [x] Life sciences
- [ ] Behavioural & social sciences
- [ ] Ecological, evolutionary & environmental sciences

For a reference copy of the document with all sections, see nature.com/documents/nr-reporting-summary-flat.pdf

Life sciences study design

All studies must disclose on these points even when the disclosure is negative.

Sample size	No sample size calculation was performed. For the cellular experiments, the sample size was determined based on common sizes used in previous publications with similar methodologies (for example: https://doi.org/10.1016/j.molcel.2019.10.035; https://doi.org/10.1038/s41467-020-15205-z; https://doi.org/10.1038/s41467-020-15119-w). For the in vitro biochemical experiments, 2-3 biological replicates were typical and widely accepted.
Data exclusions	No data was excluded from the study.
Replication	The experiments were repeated at least two times and all attempts at replication were successful.
Randomization	Additional randomization was not performed due to that the experiments were performed simultaneously and in parallel with controls.
Blinding	Blinding was not applicable in this study due to the necessity of maintaining sample labeling for analysis between groups.

Reporting for specific materials, systems and methods

We require information from authors about some types of materials, experimental systems and methods used in many studies. Here, indicate whether each material, system or method listed is relevant to your study. If you are not sure if a list item applies to your research, read the appropriate section before selecting a response.

Materials & experimental systems

n/a	Involved in the study	
☐	☒	Antibodies
☐	☒	Eukaryotic cell lines
☒	☐	Palaeontology
☒	☐	Animals and other organisms
☒	☐	Human research participants
☒	☐	Clinical data

Methods

n/a	Involved in the study	
☒	☐	ChIP-seq
☒	☐	Flow cytometry
☒	☐	MRI-based neuroimaging

Antibodies

Antibodies used	Mouse monoclonal anti-FLAG antibody, Sigma-Aldrich, Cat# F1804;<br>Mouse monoclonal anti-GFP antibody, Proteintech, Cat# 66002-1-Ig;<br>Mouse monoclonal anti-beta-actin antibody, Abcam, Cat# ab8226;<br>Goat anti-mouse secondary antibody (HRP), Abcam, Cat# ab97023;<br>Goat anti-mouse DyLight 594 IgG secondary antibody, EarthOx, Cat# E032410-01.
Validation	Anti-FLAG antibody was validated by Sigma-Aldrich (https://www.sigmaaldrich.com/catalog/product/sigma/f1804?lang=en&region=US) and used in peer-reviewed papers (PMID: 28053121, PMID: 31399583);<br>Anti-GFP antibody was validated by Proteintech (https://www.ptglab.com/products/eGFP-Antibody-66002-1-Ig.htm#validation) and used in peer-reviewed papers (PMID: 30006461, PMID: 22258766);<br>Anti-beta-actin antibody was validated by Abcam (https://www.abcam.com/beta-actin-antibody-mabcam-8226-loading-control-ab8226.pdf) and used in peer-reviewed papers (PMID: 31211445, PMID: 31168740);<br>Secondary antibody (HRP) was validated by Abcam (https://www.abcam.com/goat-mouse-igg-hl-hrp-ab97023.pdf) and used in peer-reviewed papers (PMID: 30643123, PMID: 30840666);<br>DyLight 594 IgG secondary antibody was validated by EarthOx (http://shbaiyanbio.com/upload/file/E032410.pdf) and used in peer-reviewed papers (PMID: 27643564, PMID: 23485590)

Eukaryotic cell lines

Policy information about cell lines

Cell line source(s)	HEK293T cells were purchased from American Type Culture Collection (ATCC). NRK cells with double knock out of ATG2A/ATG2B and stably transfected with GFP-LC3 were generous gifts from Dr. Li Yu (Tsinghua University).
Authentication	HEK293T cells were authenticated using STR profiling service by ATCC (https://www.atcc.org/products/all/CRL-3216.aspx#specifications). The modified NRK cells were not authenticated by us.
Mycoplasma contamination	The cell lines used in this study were tested negative for mycoplasma contamination.
Commonly misidentified lines (See ICLAC register)	None of commonly misidentified lines were used in this study.
